# Comparative Transcriptomic Reveals Greater Similarities in Response to Temperature Than to Invasive Alien Predator in the Damselfly *Ischnura elegans* Across Different Geographic Scales

**DOI:** 10.1111/eva.70002

**Published:** 2024-09-06

**Authors:** Guillaume Wos, Gemma Palomar, Marzena Marszałek, Szymon Sniegula

**Affiliations:** ^1^ Institute of Nature Conservation Polish Academy of Sciences Krakow Poland; ^2^ Department of Genetics, Physiology and Microbiology, Faculty of Biological Sciences Complutense University of Madrid Madrid Spain; ^3^ Institute of Environmental Sciences Jagiellonian University Kraków Poland

**Keywords:** comparative transcriptomics, gene expression, global warming, intraspecific variation, invasive alien species, *Ischnura elegans*

## Abstract

The impact of global changes on populations may not be necessarily uniform across a species' range. Here, we aim at comparing the phenotypic and transcriptomic response to warming and an invasive predator cue in populations across different geographic scales in the damselfly *Ischnura elegans*. We collected adult females in two ponds in southern Poland (central latitude) and two ponds in southern Sweden (high latitude). We raised their larvae in growth chambers and exposed them to combination of temperature and a predator cue released by the crayfish *Orconectes limosus*. When larvae reached the prefinal larval stage, they were phenotyped for traits related to growth and size and collected for a gene expression analysis. High‐latitude populations exhibited greater phenotypic and transcriptomic variation than central‐latitude populations. Across latitudes and ponds, temperature generally increased growth rate and the predator cue decreased mass, but the effects of temperature were also pond‐specific. Comparison of the transcriptomic profiles revealed a greater overlap in the response to temperature across latitudes and ponds, especially for pathway‐related oxidative stress and sugar and lipid metabolism. The transcriptomic response to a predator cue and to the interaction temperature × predator cue was more pond‐specific and overlapped only for few genes and pathways related to cuticle, development and signal transduction. We demonstrated that central‐ and high‐latitude populations may partially respond through similar mechanisms to warming and, to a lower extent to a predator cue and to the interaction temperature × predator cue. For the predator cue and the interaction, the large fraction of ponds‐specific genes suggests local adaptation. We show that high‐latitude populations were generally more plastic at the phenotypic and transcriptomic level and may be more capable to cope with environmental changes than their central‐latitude counterparts.

## 1 Introduction

Anthropogenic changes such as global warming, habitat degradation or biological invasions have altered functioning of ecosystems impacting animal and plant populations, and communities (Baranov et al. 2020; Strayer 2012). For organisms to persist in novel or fast‐changing environments, they have to rely on their ability to migrate, to show an immediate plastic response, and/or to respond to selective pressures through genetic changes (Bell and Collins 2008; Bellard et al. 2012). However, across a species' geographic range, the extent to which populations adapt to ongoing global changes may not be necessarily uniform and often varies along climatic clines (Dillon and Lozier 2019). This is due to the fact that life‐history traits are strongly influenced by environmental conditions, for example, temperature on growth in insects (González‐Tokman et al. 2022), leading to different life‐history strategies along environmental gradients. Hence, to better understand the species' response to environmental changes, it is important to consider multiple populations and further compare them from the phenotypic down to the molecular level (DeBiasse and Kelly 2016; Pearce‐Higgins et al. 2015).

Intraspecific variation in traits along large environmental gradients, especially along thermal clines, have been documented in many organisms. For instance, greater differentiation for growth rate and developmental time when temperature increased at higher latitudes in a damselfly (Palomar et al. 2023), larger size and faster development at higher elevations and lower temperature in beetles (Stillwell and Fox 2005) and greater differentiation in growth rate in a northern fish population along a temperature gradient (Yamahira et al. 2007). Interestingly, the response to environmental factors may also differ over short geographic distances, that is, at a local or microgeographic scale, if selection pressures are strong enough relative to gene flow. This is generally due to habitat heterogeneity and variable microclimatic conditions. Such local scale environmental variations can trigger different developmental trajectories, for example, in response to photoperiod between geographically close‐by insect populations (Lindestad et al. 2019), to temperature along a canopy gradient in amphibians (Richter‐Boix et al. 2015), and across a sand dune landscape in plants (Wos and Willi 2018). Altogether, these studies provided evidence for different sensitivity to environmental factors at different spatial scales shedding light on differences and similarities by which populations may respond to environmental changes. However, it is still unclear to what extent this variation is reflected at the molecular level and whether populations respond to similar environmental changes through similar genetic and metabolic mechanisms (Mitchell‐Olds, Willis, and Goldstein 2007; Waldvogel et al. 2020).

Gene expression variation has long been recognised as an important driver of rapid adaptation and phenotypic evolution (López‐Maury, Marguerat, and Bähler 2008). The comparison of transcriptomic profiles is a widely used approach to identify genetic underpinnings of the response to environmental variation across species or populations (Liu et al. 2017). In principle, genetic underpinnings can be detected using gene expression analysis because genes and associated metabolic pathways should exhibit expression changes in the same direction across multiple populations when exposed to similar environmental pressures (Stanford et al. 2020; Weber et al. 2015). For instance, it was demonstrated that southern damselfly populations had a lower number of genes affected by temperature than northern populations but exhibited expression changes in the same direction for genes related to mitochondria and respiratory electron transport (Swaegers, Spanier, and Stoks 2020). A fraction of genes related to development and metabolic process were differentially expressed in the same direction in response to different temperatures across multiple populations in *Drosophila* species (Huang et al. 2021; Zhao et al. 2015). Such studies were also conducted in other organisms, for example, in the scarlet monkeyflower where a large fraction of stress‐ and hormone‐related genes overlapped across populations along a latitudinal gradient in response to alternative temperatures (Preston et al. 2022). Hence, comparison of transcriptomic profiles may provide valuable insight into the magnitude and the direction of changes in gene expression or, alternatively, into conserved mechanisms or distinct responses to environmental changes.

Here, we conducted a study on the damselfly *Ischnura elegans*. We sampled populations from replicated ponds across two latitudes: southern Poland (central latitude) and southern Sweden (high latitude). We raised their larvae in growth chambers and further exposed them to two factors linked with human activities: warming and an invasive alien predator cue. Temperature and predation may impose selection on the same phenotypic traits in opposite or the same direction, for example, growth rate and mass in damselflies (Palomar et al. 2023; Amer et al. 2024) and growth rate in *Daphnia* (Tseng and O'Connor 2015). Such interactions between abiotic and biotic stressors may be an important component of adaptation of organisms to their environment; however, their impact down to the gene level has been less studied (Oliver et al. 2022). We aimed at quantifying the extent of phenotypic and transcriptomic variations in response to these two stressors across and within latitudes using phenotypic and transcriptomic data available for over a 100 individuals. A subset of these individuals was already used in a previous study focusing specifically on the transcriptomic differences between the most differentiated individuals, based on a set of phenotypic traits, under combined exposure to warming and cues from an invasive alien predator (Wos et al. 2023). Here, we proposed a larger scale study to compare the transcriptomic profiles of damselfly populations from different local ponds (microgeographic scale) and latitudes (macrogeographic scale) and to quantify to what extent they responded to temperature, an invasive alien predator and to their interaction.

For this, we used warming temperature matching the average temperature increase by the end of the century according to Intergovernmental Panel on Climate Change (Masson‐Delmotte et al. 2021) and an invasive alien predator cue released by the spiny‐cheek crayfish *Orconectes limosus*. The spiny‐cheek crayfish is currently spreading across Europe and has co‐occurred with Polish populations for several decades but has not yet been reported in Sweden (Artportalen 2024; Kouba, Petrusek, and Kozák 2014). So far, studies in *I. elegans* focused mostly on latitudinal variation of phenotypic traits in response to temperature and predator cue (Palomar et al. 2023; Stoks, Swillen, and De Block 2012; Wos et al. 2023). Few studies also indicated that variation may also occur over shorter geographic distances (Amer et al. 2024; Palomar et al. 2023; Stoks and Cordoba‐Aguilar 2012). Therefore, particular attention was paid to within‐latitude phenotypic and transcriptomic variation as such variations have rarely been explored. We specifically asked (1) Do populations from distant latitudes and different local ponds exhibit similar levels of phenotypic and transcriptomic variation in response to temperature, a predator cue and their interaction? (2) Do populations from distant latitudes and different local ponds respond to these environmental variables using similar genes and metabolic pathways?

## 2 Methods

### 2.1 Description and Sampling of *Ischnura elegans*

In our experiment, we used the common damselfly *Ischnura elegans*, a widespread species in Europe (Dijkstra and Schröter 2020). The species has variable number of generations per year (voltinism) (Corbet, Suhling, and Soendgerath 2006): at high latitudes, damselflies tend to be uni‐ and semivoltine, that is, 1 or 2 years for completing one generation, respectively (Norling 2021). At central latitudes, populations are generally uni‐ and bivoltine, that is, one or two generations per year (Corbet, Suhling, and Soendgerath 2006; Norling 2021). Within latitude, populations (hereafter, ponds) are not genetically isolated. A previous study on 10 microsatellites revealed low Fst values in *I. elegans* at central and high latitudes (Shama et al. 2011). A RADseq study based on >300,000 SNPs showed a clear genetic differentiation between central‐ and high‐latitude ponds (including three of the four ponds used in the present study, Table S1) with minor differences within each latitude suggesting strong gene flow at the local scale (Babik et al. 2023).

**FIGURE 1:**
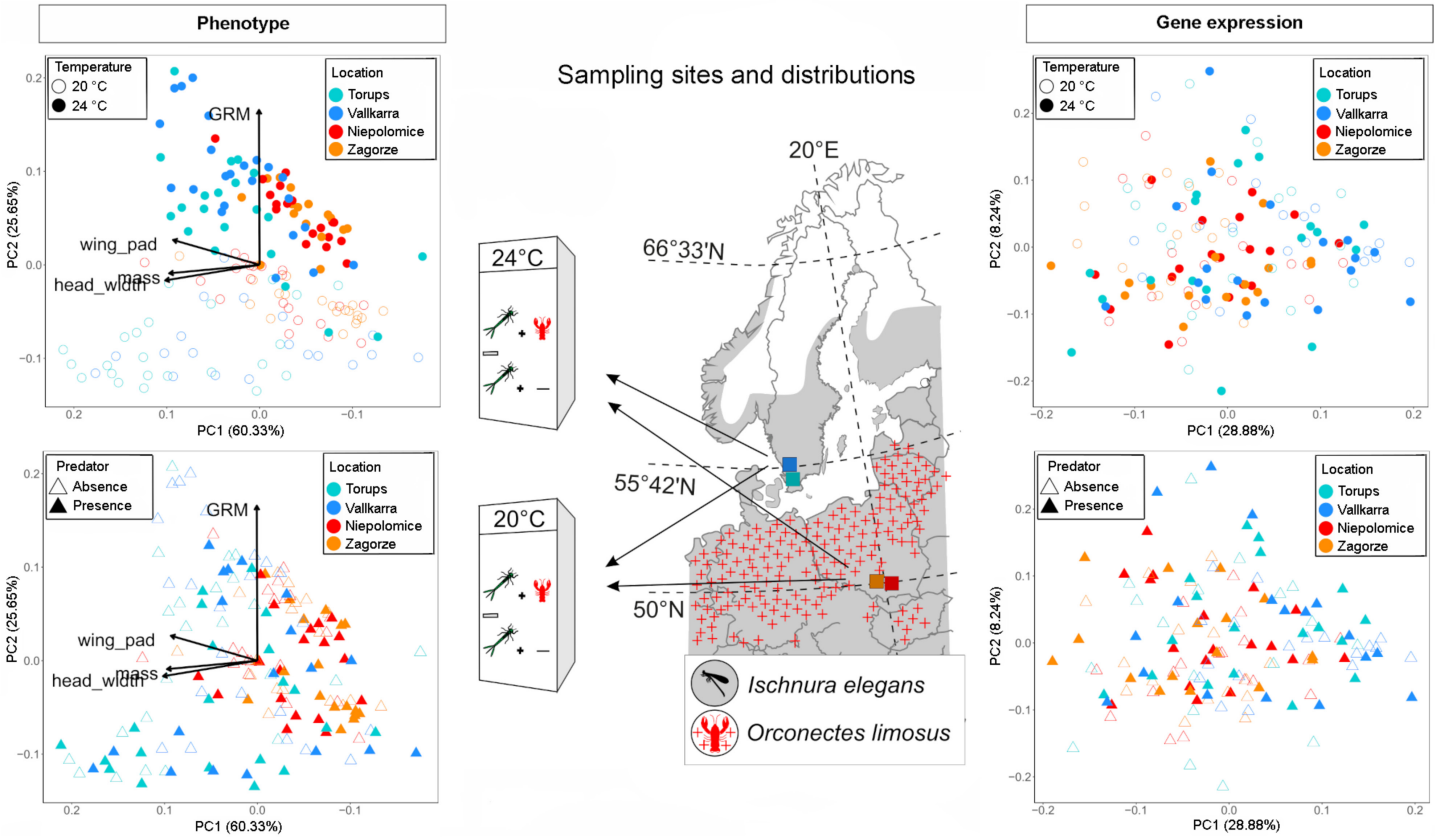
Map showing the sampled ponds depicted by coloured squares in Sweden (high latitude; blue colours) and Poland (central latitude; red colours). Larvae from field‐collected females were raised in incubators under different temperature (20°C vs. 24°C) and predator (absence vs. presence of predator cue) treatment. Geographic distribution of *I. elegans* is shown in grey (Dijkstra and Schröter 2020), and occurrence of the spiny‐cheek crayfish *O. limosus* (Kouba, Petrusek, and Kozák 2014) is depicted by red crosses. On the left side is presented principal component analysis (PCA) plots based on the phenotypic data showing differences in the four phenotypic variables between ponds and temperature (upper plot) and ponds and predator cue (lower plot). On the right side is presented PCA plots based on the transcriptomic data.

Samplings were performed as described by Wos et al. (2023). For the experiment, we collected 10 copulating tandems in two ponds at two latitudes: Niepolomice and Zagorze pond located in southern Poland (hereafter, central latitude), and Torups and Vallkarra pond located in southern Sweden (hereafter, high latitude) (Figure 1; Table S1) (2 ponds × 2 latitudes × 10 females = 40 clutches).

### 2.2 Description and Sampling of *Orconectes limosus*

The spiny‐cheek crayfish, *O. limosus*, is an invasive alien predator currently spreading in Europe (Kouba, Petrusek, and Kozák 2014). The species was introduced to central Europe at the end of the 19th century where it is considered as an old invasive alien species. It has been reported in Poland since the 1960s and its presence has not been yet detected in Scandinavia, including Sweden (Artportalen 2024; Kouba, Petrusek, and Kozák 2014). The spiny‐cheek crayfish was reported in one of our sampled ponds at central latitudes (Zagorze pond; pers. comm. with the pond owner) and is present in the Vistula river located at 150 m from the other central‐latitude pond, Niepolomice (Orłowska and Romanowski 2023).

We collected *O. limosus* from Kryspinow lake in southern Poland (50°3′0.461′′N, 19°47′20.85′′E). Crayfishes were first acclimated in laboratory conditions. For the treatment application, three crayfishes were placed in an aquarium filled with 40 L of dechlorinated tap water at a constant temperature (20°C). The density of crayfish per aquarium was determined based on previous studies that indicated the impact of crayfish cue treatment on damselfly life‐history traits (Palomar et al. 2023). For the control, we filled an aquarium with 40 L of dechlorinated tap water without animals. Crayfish collection and housing were performed with permission from the Regional Directorate for Environmental Protection in Kraków (ref. OP.672.4.2021.GZ).

### 2.3 Growth Chamber Experiment

The detailed description of the growth chamber experiment and of the selected temperatures (20°C and 24°C) are described by Wos et al. (2023); an updated version of the temperatures measured in each pond with dataloggers are presented in Figure S1. As we collected adult females at the end of June, the postwintering larval development of these individuals happened between March and June. During this time interval, water temperature reaches 20°C based on our measurements and simulations and was chosen as the control temperature. The +4°C corresponds to the predicted increased in temperatures by the end of the 21st century according to the IPCC 2021 (Masson‐Delmotte et al. 2021). The different steps of the experimental design from egg laying to the end of the experiment are also summarised in File S1. The experimental cross design involved two latitudes (high and central), two ponds, two temperatures (20°C and 24°C) and two predator treatments (presence and absence of a predator cue). We started the experiment with 10 individuals from each clutch (hereafter, maternal line) randomly assigned to one of the four treatments and placed in a separate container. This resulted in 2 latitudes × 2 ponds × 2 temperatures × 2 predator treatments × 10 maternal lines = 160 containers in total and 1600 larvae at the beginning of the experiment.

### 2.4 Life History Traits

In each container, we phenotyped the first (or two first) larvae reaching the prefinal instar before emergence (hereafter, F‐1). On the day when individuals entered F‐1, these larvae were not fed and hence were phenotyped with empty stomachs in the afternoon. In total, we measured five phenotypic traits related to larval growth and size: larval wet mass (hereafter mass; using an electronic balance Radwag AS.62), head width (proxy of structural body size in odonates; Corbet 1999), wing pad length, developmental time (number of days between hatching and entrance into F‐1) and growth rate based on mass (GRM; mass/developmental time in days). Correlation analysis conducted for each latitude separately (Table S2) revealed high correlation coefficients (*r* > 0.80 at central and *r* > 0.90 at high latitudes) between GRM and developmental time; developmental time was discarded for subsequent analyses as growth rate is generally considered a key fitness component in damselflies (Siepielski et al. 2020). We did not report the sex of each individual. However, we randomly selected larvae after egg hatching and the same procedure was applied in a previous study leading to an equal proportion of males and females (Palomar et al. 2023). After phenotyping, F‐1 individuals were preserved in RNA later and kept at −80°C for the gene expression analysis. In total, we have both phenotypic and transcriptomic data for 161 individuals (Table S3).

### 2.5 Library Preparation and Sequencing

We sequenced a total of 161 individuals from different maternal lines; however, in order to have a balance design across treatments in each pond, we only used sequenced data of 140 individuals (for Zagorze, *N* = 2 temperatures × 2 predator treatments × 8 individuals = 32; For Niepolomice, Vallkarra and Torups *N* = 2 temperatures × 2 predator treatments × 9 individuals = 36; Table S3). For each combination of pond and treatment, the eight or nine individuals sequenced belonged to different maternal lines. Except for few combinations, where two individuals among the 8 or 9 belonged to the same maternal line, however, this concerned a limited number of individuals (6 maternal lines × 2 individuals = 12 individuals in total). Library preparation and sequencing is described in details in Wos et al. (2023) where a subset of these data was used (*N* = 78). The data are available at the Sequence Read Archive under accession PRJNA899331.

### 2.6 Mapping and Differential Gene Expression Analysis

Reads were aligned on the *I. elegans* reference genome (project ID: PRJEB46264; The Darwin Tree of Life Project Consortium 2022) using hisat2 2.1.0 (Kim et al. 2019). For the alignment, we removed the sex chromosome because we did not report the sex of each individual. We counted the number of reads mapped on each gene with featurecounts 2.0.3 (Liao, Smyth, and Shi 2019), and we kept only the uniquely mapped reads (between 12.9 and 29.3 million reads per individual). Read counts were then analysed using edger 3.15.0 (Robinson, McCarthy, and Smyth 2010) in R (R Core Team 2013; RStudio Team 2015). We scaled the library size with the ‘calcNormFactors’ function, estimated dispersion using ‘estimateDisp’ and obtained, for each pond, a list of differentially expressed genes between the different treatments using the ‘glmFit’ function. For each latitude and each pond, we performed a gene expression analysis with the full model included temperature, predator and their interaction; maternal lines were also added in the model. Then, we performed three contrasts to compare the variable of interest and to obtain a list of genes affected by (1) the two temperature treatments (20°C vs. 24°C), (2) the two predator treatments (absence vs. presence of a predator cue) and (3) the interaction temperature × predator cue (T × P). We adjusted *p*‐values for multiple testing with the Benjamini and Hochberg false‐discovery rate (FDR) correction. Genes were considered as differentially expressed if FDR <0.05.

Then, for each latitude and pond, we distinguished between the genes affected only by temperature (significant effect of temperature and no significant interaction temperature × predator), only by the predator cue (significant effect of a predator cue and no significant interaction temperature × predator) and, showing significant T × P, totalling three lists of genes per latitude and pond. Next, we overlapped the different lists of genes across all latitudes and ponds in order to identify genes differentially expressed in the exact same direction in response to temperature, predator and T × P. We tested for significant intersection (*p* < 0.05) (nonrandom overlap) in gene expression using Fisher's exact test (SuperExactTest package; Wang, Zhao, and Zhang 2015).

### 2.7 Gene Ontology

For the Gene Ontology (GO) annotation, we adapted the procedure described in Wos et al. (2023). Briefly, we assigned GO terms to each *I. elegans* gene if they had a similar name and function, and were associated with the same metabolic pathways in at least two other insect species (UniProt database; Uniprot Consortium 2015). In total, we assigned GO terms to 5301 out of the 21,087 *I. elegans* genes. Then, for each gene significantly affected by temperature, predator, and by T × P, we retrieved their associated GO terms and overlapped them across ponds. We tested for significant intersection in GO terms using Fisher's exact test (SuperExactTest package; Wang, Zhao, and Zhang 2015).

### 2.8 Gene Ontology Term Enrichment Analysis

We ran a GO term enrichment analysis on the different lists of differentially expressed genes (DEGs) to identify GO terms related to biological process, cellular component and molecular function significantly over‐represented. We used BiNGO v3.0.5 software (Maere, Heymans, and Kuiper 2005) in Cytoscape v3.9.1 (Shannon et al. 2003) and the most recent gene ontology annotation (The Gene Ontology Resource; Ashburner et al. 2000). Gene information was obtained from the UniProt database (Uniprot Consortium 2015). *p*‐values were adjusted for multiple testing, and the significance level for enriched GO terms was set at FDR‐adjusted *p* < 0.05. When the number of DEGs was too small for running a GO term enrichment analysis, DEGs were just grouped according to their GO annotations.

### 2.9 Statistical Analysis

We ran PCA with the phenotypic and transcriptomic data. For the phenotypic data, we ran the PCA with the four variables: mass, head width, wing pad and GRM; variables were scaled and centred before running the PCA. For the transcriptomic data, we calculated transcript per million (TPM) values for each gene with detectable expression (*N* = 11,748) before running the PCA. Then, we used permutational analysis of variance (PERMANOVA; adonis2 function in vegan package; Oksanen et al. 2013) to test for the effects of (1) latitude, pond, temperature, predator and the following interaction: temperature × predator cue, temperature × pond, predator cue × pond and temperature × predator cue × pond on the complete phenotypic and transcriptomic dataset and (2) temperature for each pond separately. All statistical analyses were done in R (R Core Team 2013; RStudio Team 2015).

For both phenotypic and transcriptomic data, we also quantified the range of phenotypic and transcriptomic variation between latitudes and ponds using disparity analysis (R package disparity Guillerme 2018). We calculated disparity as the median distance between each individual and their centroid of the corresponding latitude or pond in the ordination space. Median distance and confidence intervals were derived from 1000 bootstraps replicates. Significance was tested using the adonis2 function implemented in the package disparity (function adonis.dispRity). The model included the predictors affecting the most the phenotypic and transcriptomic data as detected by PERMANOVA: latitude, temperature, predator cue, pond and temperature × pond.

## 3 Results

### 3.1 Effects of Geographic Origin and Treatments on the Phenotype

At the phenotypic level, PCA plots and PERMANOVA test showed significant effects of the three variables latitude, temperature and predator cue on the set of phenotypic traits (Table 1A; Figure 1). Latitude explained the greatest proportion of variance (32%), followed by temperature (4%) and predator cue (4%) (Table 1A). The direction of the phenotypic changes involved higher mass, larger head width, and longer wing pad at high latitudes (Table S4). Lower GRM was observed at 20°C compared to 24°C (Table S4). There were significant effects of pond and of the interaction temperature × pond on the phenotype explaining 3% of the variance, and we further looked at these pond and temperature‐specific effects.

**TABLE 1:** Permutational analysis of variance (PERMANOVA) to test for the effects of latitude, pond, temperature, predator cue and some of their interactions on (A) the combination of the four phenotypic traits (mass, head width, wing pad length and growth rate based on mass [GRM]) and (B) the transcriptome.

		(A) Phenotype		(B) Gene expression	
Variables	df	*R* ^2^	*p* (*F*)	*R* ^2^	*p* (*F*)
Latitude	1	**0.32**	**<0.001 (91.1)** ***	**0.03**	**0.001 (5.06)** ***
Temperature	1	**0.04**	**<0.001 (11.3)** ***	**0.05**	**0.001 (8.31)** ***
Predator cue	1	**0.04**	**0.002 (11.7)** **	**0.02**	**0.008 (3.40)** **
Pond	2	**0.03**	**0.008 (4.98)** **	0.01	0.513 (0.90)
Temperature × predator cue	1	0.01	0.073 (3.36)	0.00	0.686 (0.621)
Temperature × pond	3	**0.03**	**0.023 (3.08)** *	**0.03**	**0.054 (1.65)** *
Predator cue × pond	3	0.00	0.901 (0.22)	0.02	0.490 (0.97)
Temperature × predator cue × pond	3	0.02	0.148 (1.83)	0.01	0.807 (0.70)

*Note:* Significance is indicated in bold.

Abbreviations: df, degree of freedom; *R* ^2^, percentage of variance explained.

*** *p* < 0.001.

** *p* < 0.01.

* *p* < 0.10.

When looking at each pond separately, temperature had significant effects on the phenotype in one central‐ and one high‐latitude ponds, Niepolomice and Torups, explaining 19% and 16% of the total variance, respectively (Table 2A; Figure S2). Although the effect of temperature was similar across the four ponds for GRM (Table S4), the other variables showed pond‐specific effects. In Torups and Niepolomice ponds, results showed significant differences in mass between the two temperature treatments with higher mass at 20°C. In Torups only, head width was larger at 20°C (Table S4). Finally, in the two high‐latitude ponds, we observed effects of temperature on wing pad length but going in different directions; with longer wing pad at 20°C in Torups and at 24°C in Vallkarra.

**TABLE 2:** Permutational analysis of variance (PERMANOVA) to test for the effects of temperature on (A) the four phenotypic traits (mass, head width, wing pad length and growth rate based on mass [GRM]) and (B) the transcriptome for each pond separately.

		Central latitude				High latitude			
		Zagorze		Niepolomice		Vallkarra		Torups	
	df	*R* ^2^	*p* (F)	*R* ^2^	*p* (F)	*R* ^2^	*p* (F)	*R* ^2^	*p* (F)
(A) Phenotype									
Temperature	1	0.03	0.303 (1.17)	**0.19**	**0.006 (8.88)** **	0.02	0.324 (1.01)	**0.16**	**0.008 (8.27)** **
(B) Gene expression									
Temperature	1	**0.07**	**0.038 (2.32)** *	0.05	0.100 (1.97)	**0.10**	**0.002 (3.72)** **	**0.11**	**0.002 (4.39)** **

*Note:* Significance is indicated in bold.

Abbreviations: df, degree of freedom; *R* ^2^, percentage of variance explained.

** *p* < 0.01.

* *p* < 0.05.

**FIGURE 2:**
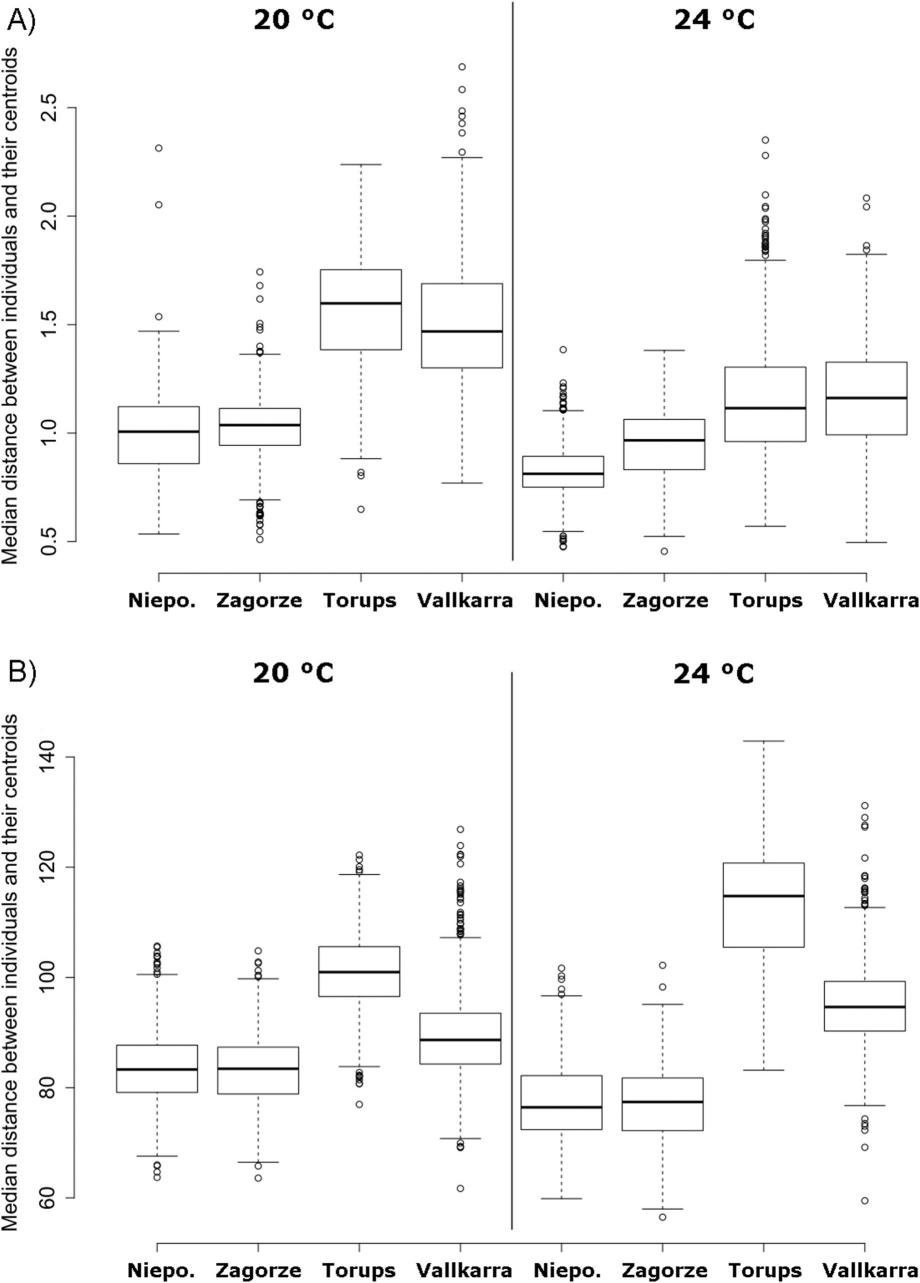
Boxplots of the median distance between individuals and their centroids for each pond and temperature treatment based on principal component analysis (PCA) plots using (A) the phenotypic and (B) the transcriptomic data. Median and 95% confidence intervals were derived from 1000 bootstraps replicates. Statistics are presented in Table 3.

Phenotypic differentiation, as measured by disparity, was significantly higher in the two high‐latitude ponds at both 20°C and 24°C indicating that individuals were more similar to each other at central compared with high‐latitude ponds (Figure 2; Table 3). The interaction temperature × pond was significant, with a greater difference between ponds at 20°C than at 24°C.

**TABLE 3:** Disparity analysis testing for the effects of latitude, temperature, predator cue, pond and the interaction temperature × pond on the median distance between individuals and their centroids shown in Figure 2 for (A) the phenotypic and (B) the transcriptomic data.

		(A) Phenotype		(B) Gene expression	
Variables	df	*R* ^2^	*p* (*F*)	*R* ^2^	*p* (*F*)
Latitude	1	**0.15**	**<0.001 (40.9)** ***	**0.05**	**<0.001 (7.96)** ***
Temperature	1	**0.17**	**<0.001 (8.93)** ***	**0.02**	**0.013 (2.59)** *
Predator cue	1	**0.03**	**<0.001 (4.56)** ***	**0.01**	0.202 (1.23)
Pond	2	**0.04**	**<0.001 (46.9)** ***	**0.02**	**0.021 (1.82)** *
Temperature × pond	3	**0.05**	**<0.001 (4.46)** ***	**0.02**	0.220 (1.16)

*Note:* Significance is indicated in bold.

Abbreviations: df, degree of freedom; *R* ^2^, percentage of variance explained.

*** *p* < 0.001.

* *p* < 0.05.

### 3.2 Effects of Geographic Origin and Treatments on the Transcriptome

At the whole‐transcriptomic level, there were significant effects of the three variables latitude, temperature and predator cue on the transcriptome (Figure 1; Table 1B). Temperature explained the greatest proportion of transcriptomic variance (5%), followed by latitude (3%) and predator cue (2%). The interaction temperature × pond was at a boarder of significance (*p* = 0.054). Other interactions were not significant. When looking at each pond separately, we observed, as a trend, stronger effects of temperature in term of variance explained on the two high‐latitude ponds Torups (11%) and Vallkarra (10%) (Table 2B; Figure S3). At central latitude, temperature had a significant effect only in Zagorze pond (7% of explained variance).

Transcriptomic differentiation, as measured by disparity, was significantly higher in high‐latitude ponds at both 20°C and 24°C indicating that high‐latitude individuals harboured greater variation in their gene expression values compared with central‐latitude individuals (Figure 2; Table 3). However, this pattern was mostly driven by the high‐latitude pond Torups in which the median distance between individuals and their centroids was higher than in the other ponds (Figure 2).

### 3.3 Similarity in Gene Expression Across Latitudes

For each latitude separately, we distinguished between the genes showing significant effects of temperature, predator cue and T × P. We tested to what extent the genes affected by these treatments were differentially expressed in the same direction across central and high‐latitude individuals.

**FIGURE 3:**
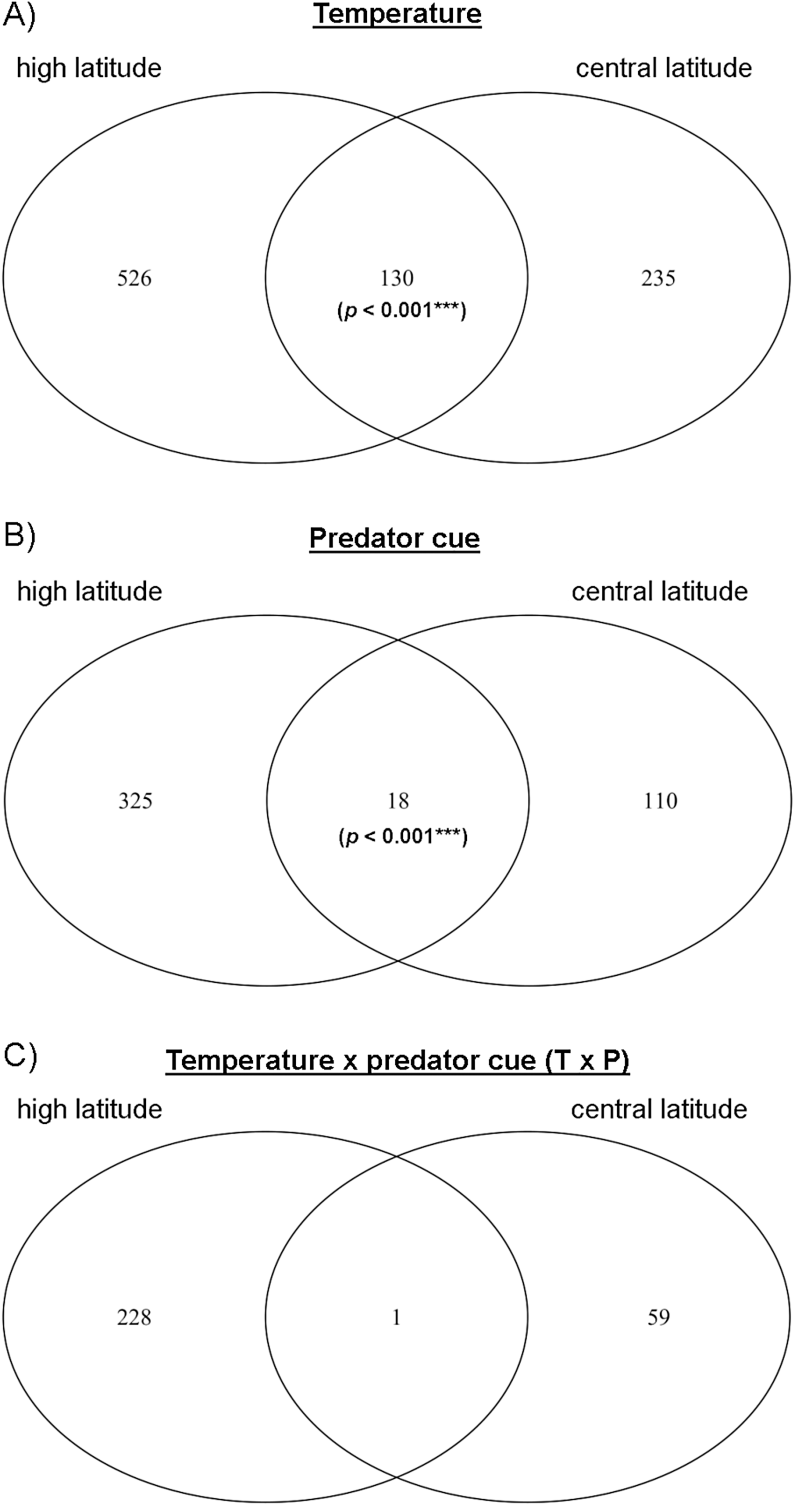
Venn diagrams showing the number of differentially expressed genes (DEGs) in response to (A) temperature, (B) predator cue and (C) interaction temperature × predator cue (T × P) at each latitude and their overlap. The overlap indicates the number of genes differentially expressed in the same direction across the two latitudes. Significance (nonrandom overlap) was assessed using Fisher's exact test.

We observed different transcriptomic patterns with central‐latitude populations being quantitatively less affected by the treatments than high‐latitude populations as fewer genes were differentially expressed (Figure 3). By overlapping the list of DEGs, we found that the number of genes differentially expressed in the same direction between central and high latitude was significantly greater than expected by chance in response to temperature (130 genes) and to the predator cue (18 genes) (Table S5). For the temperature treatment, the GO term enrichment analysis on the 130 overlapping genes revealed mostly GO terms related to the response to oxidative stress, defence system, protein complex assembly and protein localisation. For the predator treatment, no GO term was significantly enriched, only two of the 18 are described: *PROTON‐COUPLED AMINO ACID TRANSPORTER 1* (LOC124158508) and *CHONDROITIN PROTEOGLYCAN‐2‐LIKE* (LOC124159874).

### 3.4 Similarity in Gene Expression Across Ponds

**FIGURE 4:**
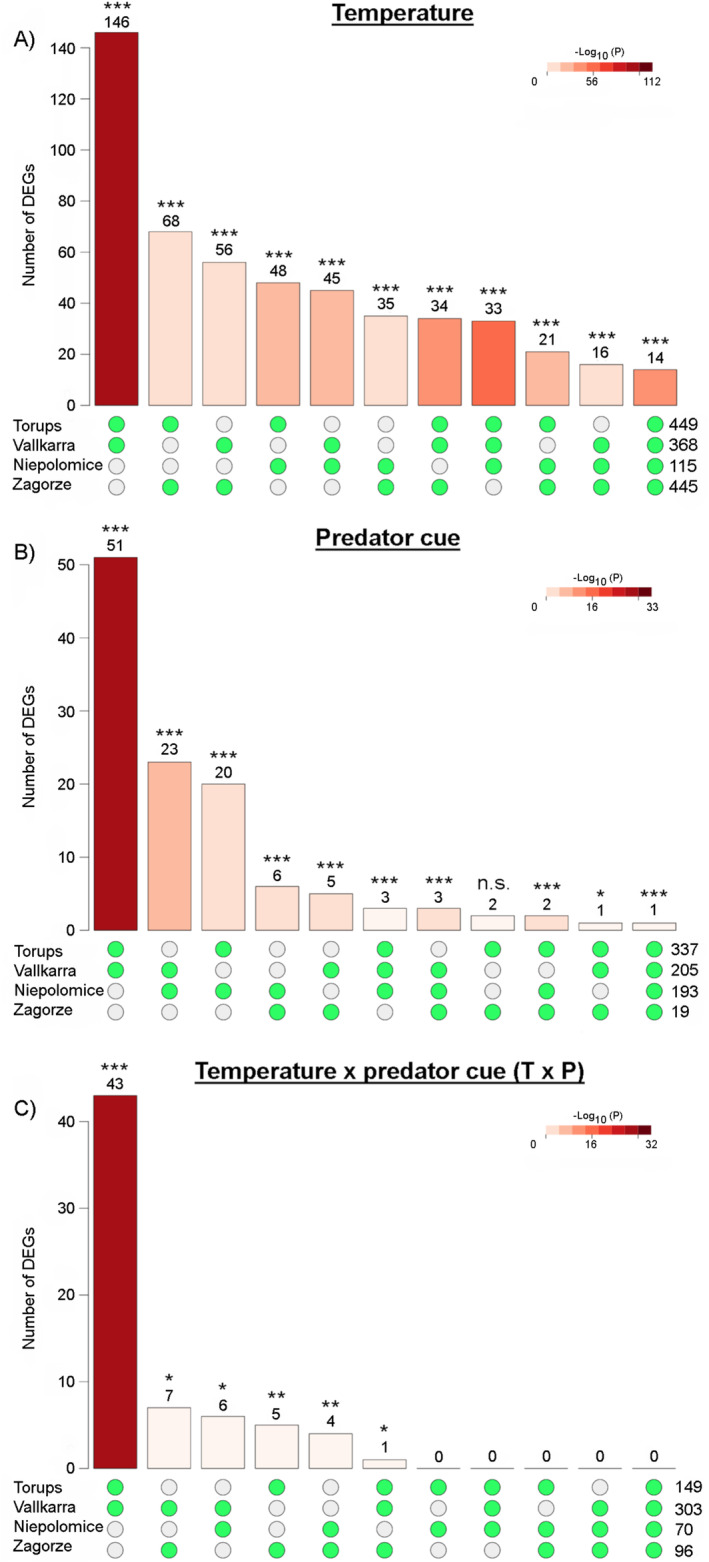
Number of differentially expressed genes (DEGs) in the same direction across ponds in response to (A) temperature, (B) predator cue and (C) interaction temperature × predator cue (T × P). On the *x*‐axis, green dots indicate the ponds being compared and values on the right side indicate the total number of genes affected by each treatment in each pond. Significant overlap across ponds was assessed by Fisher's exact test and is indicated by ***p < 0.001, **p < 0.01, *p < 0.05 and nonsignificant results are indicated by n.s.; detailed results of the tests are presented in Table S7.

Next, we looked at the overlap across ponds (Figure 4). For the response to temperature, a total of 232 genes were differentially expressed in the same direction across at least two ponds (Table S6). The number of DEGs in each intersection was significantly greater than expected by chance indicating a shared response to temperature across all ponds, with the greatest overlap found between the two high‐latitude ponds (Table S7). We found that 14 genes were differentially expressed in the same direction across the four ponds, and three of them were described: *SALIVARY GLUE PROTEIN SGS‐3‐LIKE* (LOC124163034; Figure S4), *NEUROGENIC LOCUS PROTEIN DELTA‐LIKE* (LOC124154428) and *PEROXIDASE‐LIKE* (LOC124156868) genes. We ran a GO term enrichment analysis on these 232 genes, we found two enriched GO terms for cellular component; with gene products located in extra cellular regions and 13 for molecular function related to vitamin‐, haem‐ and chitin‐binding activity, and to serine‐peptidase activity.

For the response to a predator cue, 92 genes were differentially expressed in the same direction across at least two ponds. The number of DEGs was significantly greater than expected by chance in all but one case (Tables S6 and S7). The greatest overlap was found between the two high‐latitude ponds followed by the overlap between Vallkarra ꓵ Niepolomice (illustrative example in Figure S5) and Torups ꓵ Niepolomice, whereas the overlap between the two central‐latitude ponds contained only a small number of genes in comparison. Only one gene was differentially expressed in the same direction across the four ponds but its function was not known. The GO term enrichment analysis on the 92 genes revealed 12 enriched GO terms for biological process and was part of phosphate and anion transport and of defence and response to other organisms. Finally, six GO terms for molecular function related to pigment binding, lipase and transport activity.

For the response to T × P, a total of 63 DEGs show significant interaction in the same direction across at least two ponds reflecting constitutive differences in the plastic response. The number of DEGs was significantly greater than expected by chance in all intersections (Tables S6 and S7). The greatest overlap was found between the two high‐latitude ponds and only one gene overlap across three ponds (*A‐KINASE ANCHOR PROTEIN 14‐LIKE* [LOC124169622]; Figure S6). We found 20 enriched GO terms for biological process and five for cellular function due to one gene *PROTEIN MASQUERADE* (LOC124164068; Table S6) involved in central nervous system and axon development. Enriched GO terms for molecular function were related to cuticle formation.

### 3.5 Similarity in GO Terms Between Ponds

Apart for similarities in gene expression, we also tested for significant overlaps in metabolic pathways and gene functions. For this, we retrieved for each gene differentially expressed in the same direction across at least two ponds their respective GO terms and overlapped them (Figure S7; Table S8). For all treatments, all intersections were significant.

For the response to temperature, 31 GO terms overlap across the four ponds and were related to various functions such as anatomy, sugar and lipid metabolism, cell organisation or oxidative stress.

For the response to the predator cue, two GO terms overlapped across all ponds: integral component of membrane and oxidoreductase activity and 19 GO terms overlapped across three ponds and were related to cuticle formation, calcium binding, sugar and lipid metabolism or neuropeptide signalling.

Finally, for the response to the interaction temperature × predator, five GO terms overlapped across all ponds and were associated with kinase and endopeptidase activity and structural constituent of cuticle.

## 4 Discussion

We aimed at comparing phenotypic and transcriptomic variation in response to multiple stressors in the damselfly *I. elegans* within and across latitudes. Our results showed greater phenotypic and transcriptomic variability at high‐ compared with central latitudes, suggesting higher adaptive potential at high latitudes. We also found a greater overlap in the transcriptomic profiles in response to temperature compared with that of the predator cue, especially for pathways related to oxidative stress, protein turnover and metabolism. On the contrary, transcriptomic profiles in response to a predator cue were more latitude and pond‐specific, and the small fraction of overlapping genes was related to defence mechanisms and metabolism. Similarly, for the response to the interaction between temperature and predator cue (T × P), the small fraction of overlapping genes was involved in cuticle formation or signalling pathways, indicating that the crayfish predator, despite being present only at central latitudes, may induce a shared response across all ponds.

### 4.1 Phenotypic Response

At the phenotypic level, our results indicated a stronger effect of latitude compared with temperature and predator cue. Latitudinal effects were manifested by slower growth at high latitudes and at 20°C, and lower mass in the presence of a predator cue. The damselfly response in phenotypic traits confirmed previous findings (Śniegula, Johansson, and Nilsson‐Örtman 2012; Stoks, Swillen, and De Block 2012). The latitudinal differences may be attributed to variable voltinism induced by different thermo‐photoperiod leading to different life‐history strategies. Central populations produce on average one more generation per year than high‐latitude populations (Corbet, Suhling, and Soendgerath 2006; Norling 2021) and develop and grow faster with a lower mass (Stoks, Swillen, and De Block 2012). Apart from latitude, temperature and a predator cue had considerably weaker but still significant effects on the phenotype. Metabolism in ectotherms is strongly temperature dependent and an increase in temperature, but until a certain temperature threshold (Frazier, Huey, and Berrigan 2006) is associated with an increase in growth rate (Palomar et al. 2023; Stoks, Swillen, and De Block 2012). Previous works demonstrated an effect of a predator cue on mass of *I. elegans* independently of their latitude of origin (Palomar et al. 2023). Such response might be explained by the presence of other crayfish species, that is, noble crayfish (*Astacus astacus*) present at both studied latitudes (Kouba, Petrusek, and Kozák 2014), which might enable predator cue recognition (Anton et al. 2020).

We also found variation within latitudes for the set of phenotypic traits but only in response to temperature. Variation in size related traits at a local scale was already reported in damselflies for head width (Śniegula, Nilsson‐Örtman, and Johansson 2012) and also in other insects, for example in water striders (Blanckenhorn 1991). Such variations over relatively short spatial scales are generally due to habitat heterogeneity, or here to pond topography, creating different microclimatic conditions (Pincebourde et al. 2016). Hence, different environmental conditions manifested as different selection pressures may lead to locally adapted populations despite high levels of gene flow (Richardson et al. 2014). In our study, we observed subtle differences in temperatures between our sampled ponds at a given latitude (Figure S1). Such small differences in temperature might partially shaped damselfly traits, as shown in another study on *I. elegans* (Amer et al. 2024). We cannot exclude variations in other biotic and abiotic factors between our sampled ponds that may also affect damselfly growth and size, that is, population density and intra‐ and interspecific competition (Raczyński et al. 2021; Sniegula, Golab, and Johansson 2017).

Another explanation for the reported phenotypic difference at a local scale may be related to asynchronous phenology in terms of emergence and breeding dates and variable voltinism within latitudes (Norling 2021). Previous studies conducted on a confamiliar damselfly *Coenagrion pulchellum* (Śniegula, Johansson, and Nilsson‐Örtman 2012) and the butterfly *Pararge aegeria* (Lindestad et al. 2019) with variable voltinism demonstrated that local populations had different sensitivity to the thermo‐photoperiod with downstream effects on their development rate and associated voltinism.

Our disparity analysis revealed greater phenotypic variation at high than at central latitudes with smaller differences within latitude. Similar trends in disparity were also found at the transcriptomic level, which is discussed below. Theories predict that high levels of thermal plasticity tend to be favoured in more variable environments assuming sufficient genetic variation (Leung et al. 2020; López‐Maury, Marguerat, and Bähler 2008). The theory was supported empirically in insects (Morgan Fleming, Carter, and Sheldon 2021) and plants (Richter et al. 2012) at the phenotypic level and in beetles (Belén Arias, Josefina Poupin, and Lardies 2011) and corals (Kenkel and Matz 2016) at gene expression level. Here, we cannot confirm that high‐latitude ponds are more variable than the central‐latitude ones even though environments, especially thermal conditions, tend to become more variable towards higher temperate latitudes (Louthan, DeMarche, and Shoemaker 2021). In addition, it was predicted that greater among‐year (or among‐generation) thermal variation, as found in high‐latitude environments, induced high phenotypic differentiation for growth rate in several damselfly species, whereas the opposite pattern was observed at lower latitudes where the among‐generation thermal variation is lower (Nilsson‐Örtman et al. 2012). In our study, the latitudinal distance between high‐ and central‐latitude ponds is not large (five degree difference in latitude and similar elevations) making it difficult to explain the greater phenotypic and transcriptomic variation of the high‐latitude ponds solely by environmental and thermal variability. A previous genetic study including some of our sampled ponds showed a lower genetic diversity in southern Poland than in southern Sweden (Babik et al. 2023), which was consistent with our findings of a lower phenotypic variation in central‐ than high‐latitude ponds. Further research that considers environmental variation over time and space might help explaining patterns of phenotypic differentiation.

### 4.2 Gene Expression

Next, we compared gene expression variations across latitudes and ponds in response to temperature and a predator cue at the whole‐transcriptomic level and on a per‐locus basis.

At the whole‐transcriptomic level, the proportion of variance explained by latitude differed considerably compared with our observations at the phenotypic level. The strong latitudinal effects observed at the phenotypic level were not necessarily reflected at the whole‐transcriptomic level and may indicate that latitude affects a limited number of genes (probably of large effects) rather than the entire transcriptome. At the transcriptomic level, the contribution of latitude, temperature and predator cue tended to be of the same order of magnitude, with temperature explaining a slightly greater proportion of variance than latitude and predator. We also noted that the number of genes affected by the two treatments: temperature and predator cue, was quantitatively higher at high than central latitudes and exhibited greater variability in gene expression levels. These results supported a previous study on *I. elegans* that showed a greater transcriptomic response to temperature in high‐latitude than in low‐latitude populations (Swaegers, Spanier, and Stoks 2020). Our work also agreed with the general assumption that nonadapted populations when facing novel environmental conditions (here the spiny‐cheek crayfish at high latitudes) exhibited a stronger transcriptomic response than adapted populations (Franssen et al. 2011). Finally, no interactions between the two treatments and ponds were detected, except a barely significant interaction between temperature and pond. This may be due to, first, the relatively low number of genes, but still biologically meaningful, affected by the treatments (especially the predator cue) compared with the total number of genes expressed and, second, a relatively low number of genes that probably differ between ponds.

Comparisons of the transcriptomic profiles on a per‐locus basis revealed a variable number of genes affected by temperature, predator cue and T × P across ponds suggesting that the magnitude of the response to novel environmental conditions was not uniform within a latitude that may be due to variable local abiotic and biotic conditions within each pond.

Temperature is an important determinant of growth and development in ectotherms; therefore, pathways related to development and metabolism are more likely to be conserved across latitudes and ponds (González‐Tokman et al. 2022). Latitudinal comparison of transcriptomic profiles in response to temperature revealed mostly pathways related to oxidative stress and protein assembly. In general, changes in cellular metabolism and increase in oxygen consumption, often associated with faster growth rate, lead to accumulation of reactive oxygen species (Monaghan, Metcalfe, and Torres 2009) and a rapid protein turnover or protein synthesis is a common response to an increase in temperature as previously shown in *I. elegans* (Swaegers, Spanier, and Stoks 2020). Another study on *I. elegans* found that heat‐shock proteins and genes involved in the neural system were conserved across the species' range in response to different temperatures (Lancaster et al. 2016). However, in this latter study, the authors used considerably higher air temperature (43°C) rather than mild warming temperatures on adult damselflies. Across ponds, we also observed high degree of conservation at both the gene and GO term level and some of them were related to development and metabolism (sugar and lipid metabolism) which was consistent across experimental temperatures. At the gene level, genes showing differential expression across the four ponds were shown to be related to development and metamorphosis (*SALIVARY GLUE PROTEIN SGS‐3‐LIKE*) (Da Lage et al. 2019) and neurogenesis (*NEUROGENIC LOCUS PROTEIN DELTA‐LIKE*) (Kopczynski et al. 1988) in *Drosophila*. We also identified more specific pathways that overlapped across the four ponds, that is, related to serine or vitamin binding activity, previously shown to be also affected by heat in other insect species (Liu et al. 2017; Robert Michaud et al. 2008). Proteins of the serine family have putative functions in immune response and phenoloxidase activation related to oxidative stress (González‐Santoyo and Córdoba‐Aguilar 2012).

For the response to the predator cue, we observed great variability in the number of DEGs across latitudes and ponds. We hypothesise that long‐term coexistence with the predator at central latitudes may mitigate the transcriptomic response especially in the Zagorze pond in which a very weak signal was detected (only 19 DEGs). Despite significant overlap, the response to predator appeared to be more specific even between central‐latitude ponds. This may be due to the fact that the two central‐latitude ponds may have different history with the predator, for example, Niepolomice pond is older than Zagorze pond. Latitudinal comparison revealed a significant overlap but only with two genes described with the current knowledge: *PROTON‐COUPLED AMINO ACID TRANSPORTER 1* putative role in growth amino acid transport and *CHONDROITIN PROTEOGLYCAN‐2‐LIKE* chitin‐binding activity. However, the link between these two genes with biotic stress was not very clear. Across ponds, little overlap was found at the gene level and only for genes involved in defence mechanisms and phosphate ion transport which may have various functions from energy production to cellular signalling. Similarly, only a few GO terms overlapped and were related to cuticle formation, calcium binding, sugar and lipid metabolism, and neuropeptide signalling. This was consistent with the growth reduction and potential changes in metabolism associated with the predator cue. The neuropeptide signalling pathway regulates number of physiological and behavioral functions in animals (Elphick, Mirabeau, and Larhammar 2018). In insects, the cuticle has many different functions, that is, resistance to abiotic and biotic stressors, sensory or body protection (Vincent and Wegst 2004), and is likely to be involved in resistance against predators.

For the T × P, no significant overlap was found between latitudes. Across ponds, despite a similar phenotypic response (absence of significant three‐way interaction temperature × predator cue × pond), comparisons of the transcriptomic profiles indicated that the response seemed to involve partly the same pathways but not necessarily the exact same genes as the overlap was more important at the GO term level. Hence, similar phenotypes in response to interacting factors may be achieved using different sets of genes, as previously shown in *Daphnia* in response to temperature and predator (Oliver et al. 2022). Only one gene overlapped across three ponds (*A‐KINASE ANCHOR PROTEIN 14‐LIKE*) which was probably involved in signal transduction and enzyme activation as proteins kinase A are ubiquitous signalling proteins. Another gene responsible of many enriched GO terms (*MASQUERADE PROTEIN*) is involved in central nervous system and axon development and potentially in sensory mechanisms (Murugasu‐Oei et al. 1996). Overlapping GO terms across the four ponds were associated with cuticle formation, and with endopeptidase and kinase activity that have a role in signal transduction. To summarise, we found differences in the absolute number of genes and in the overlap across latitudes and ponds in response to T × P compared with each stressor applied separately. This tended to indicate that T × P represented a distinct stress triggering a distinct transcriptomic response. Furthermore, it was demonstrated in *Daphnia* that the transcriptomic response to the interaction between temperature and predator may be even genotype‐dependent (Oliver et al. 2022) suggesting a role of the evolutionary history of each genotype (or maternal line) in shaping the response to interacting stressors. Our experimental design allowed only latitude and pond comparison, as we were lacking replicates for each maternal line. But the response to interacting stressors appeared to be less uniform across latitudes and ponds than the response to each stressor applied separately.

Finally, our gene expression results pointed to greater variability in response to treatments in high‐ than in central‐latitude ponds. These results were consistent with greater genetic variations found in these populations (Babik et al. 2023). This may reflect different levels of transcriptomic plasticity, and it has been proposed that genotypes that exhibit high level of plasticity in response to novel environmental conditions may be more likely to survive compared with less‐plastic genotypes (Lohman, Stutz, and Bolnick 2017). Indeed, genetic variation for plasticity may provide the raw material for further selection and adaptation (Schlichting and Pigliucci 1998). It is also worth noting that high‐latitude populations were numerous and situated far from the margins of the species' geographic range, minimising the effects of genetic drift, or preventing steeper selection gradients that are expected at range margins, as shown in previous studies (Eckert, Samis, and Lougheed 2008; Sniegula et al. 2016). Altogether, our results suggested that high‐latitude populations may be more capable to cope with environmental changes than their central‐latitude counterparts.

## 5 Conclusion

We demonstrated that geographically different populations may respond to mild warming temperature through similar mechanisms at the gene and metabolic pathway levels, and to a lower extent to an invasive alien predator stress. Hence, our work highlighted the transcriptomic differences in coping with abiotic and biotic stressors involving different mechanisms and metabolic pathways that may ultimately pose different challenges for organisms, especially if these abiotic and biotic stressors interact. For the interacting effect of temperature and predator cue, comparison of transcriptomic profiles indicated that the response tended to be more latitude‐ and pond‐specific that might be explained by the different life‐history strategies related to voltinism and to pond‐specific characteristics. Our results suggest differences in the adaptive potential of studied populations to environmental changes, particularly in high‐latitude populations which expressed a greater variability in their response at the phenotypic and transcriptomic levels, even when facing novel sources of disturbance such as unfamiliar predator stress. As we studied the immediate response to environmental changes, further works are needed to investigate how this may contribute to long‐term adaptation to global changes induced by human activities.

## Conflicts of Interest

The authors declare no conflicts of interest.

## Supporting information


**File S1.** Summary of the different steps of the growth chamber experiment.
**Figure S1.** Weekly temperatures in each pond.
**Figure S2.** Principal component analysis with the phenotypic data.
**Figure S3.** Principal component analysis with the transcriptomic data.
**Figure S4.** Expression values for the gene SALIVARY GLUE PROTEIN SGS‐3‐LIKE.
**Figure S5.** Expression values for the gene AGRIN.
**Figure S6.** Expression values for the gene A‐KINASE ANCHOR PROTEIN 14‐LIKE.
**Figure S7.** Overlap in gene ontology terms.
**Table S1.** Information about sampling sites.
**Table S2.** Spearman correlation analysis.

**Table S3.** Dataset used in this study.

**Table S4.** Pairwise comparisons.

**Table S5.** Number of genes differentially expressed in the same direction across the two latitudes and for each treatment.

**Table S6.** Number of genes differentially expressed in the same direction across at least the two ponds and for each treatment.

**Table S7.** Results of the Fischer’s Exact test for the significance of each overlap across ponds and for each treatment at the gene level.

**Table S8.** Results of the Fischer’s Exact test for the significance of each overlap across ponds and for each treatment at the GO term level.

## Data Availability

The RNA‐seq data used in this study are available at Sequence Read Archives (project ID PRJNA899331). The phenotypic data are included in Table S3 of the present study.
