# Supplementary material for: Comparative Transcriptomic Reveals Greater Similarities in Response to Temperature Than to Invasive Alien Predator in the Damselfly Ischnura elegans Across Different Geographic Scales
Source: Evol Appl. 2024 Sep 6;17(9):e70002. doi: 10.1111/eva.70002 (PMC11377989; doi:10.1111/eva.70002)

**Supplementary information**

**Comparative transcriptomic reveals greater similarities in response to temperature than to invasive alien predator in the damselfly *Ischnura elegans* across different geographic scales**

**File S1. Methods**

*Growth chamber experiment*

Summary of the different steps of the growth chamber experiment. A more detailed description of the methods is available in Wos et al. (2023). Abbreviations: L:D – light:dark, F-1 – pre-final instar before emergence.

| **Step** | **Conditions** | **Procedure** |
| --- | --- | --- |
| **Egg laying** | 22°C and natural daylight (end of June); approx. 3 days | Each adult female was placed in individual plastic box with wet filter paper for egg laying |
| **Egg incubation** | 22 °C; L:D 20:4 h; approx. 2 weeks | Each egg clutch (hereafter, maternal line) was placed in a separate container filled with water |
| **Egg hatching** | 22 °C; L:D 20:4 h; approx. 2 days | 10 individuals from each maternal line were randomly assigned to one of the four treatments. These 10 individuals were reared in groups, as it happens in nature, in new containers filled with water |
| **Treatment application** | 20 °C or 24 °C; L:D 20:4 h; presence or absence of predator cues; 1 – 5 months (until the first larvae entered instar F-1) | Containers were transferred to separate incubators corresponding to the two temperature treatments. At the same time, initiation of the predator treatment by refilling a third of the container with water either from the crayfish or from the control aquarium. Water was refilled every two days.  Feeding: *Ad libitum* with *Artemia* nauplii, twice a day (week days) and once a day (weekend days) |
| **End of the experiment** | 20 °C or 24 °C; L:D 20:4 h; presence or absence of predator cues | In each container, the first larva reaching F-1 was collected for phenotyping and the gene expression analysis. When two larvae reached F-1 the same day, we collected the two larvae to increase the sample size. |

**Table S1**. Information about sampling sites including elevation a.s.l., GPS coordinates, surface of each pond, percentage of impervious surface (proxy of land transformation and urbanization level), visual assessment of tree coverage of the shore of each pond in percentage (tree cover) and genetic diversity estimates. Genetic diversity estimates were retrieved from a previous study (Babik et al. 2023) and were available for three or the four ponds (Niepolomice, Zagorze and Vallkarra). For Torups, we reported the genetic diversity estimate of a nearby pond located at 1.3 km away from the Torups pond (pond “Se_S_R2”in Babik et al. 2023).

| **Country** | **Locality** | **Latitude** | **Pictures** | **Elevation (m)** |
| --- | --- | --- | --- | --- |
| Poland | Niepolomice | Central | 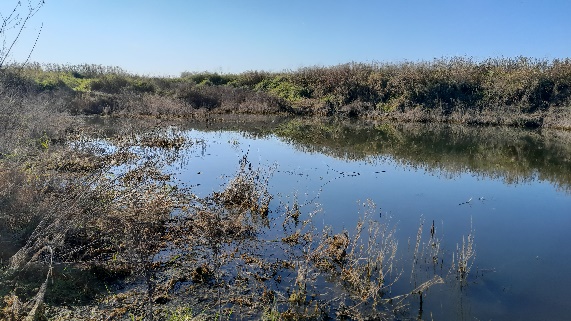 | 192 |
| Poland | Zagorze | Central | 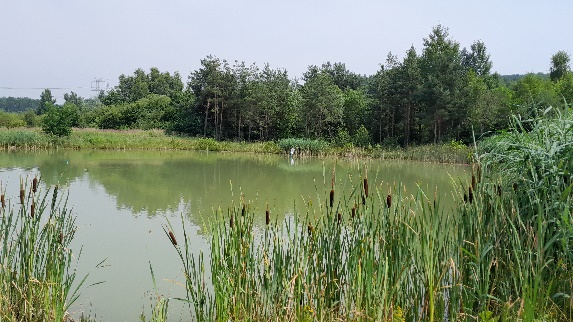 | 275 |
| Sweden | Torups | High | 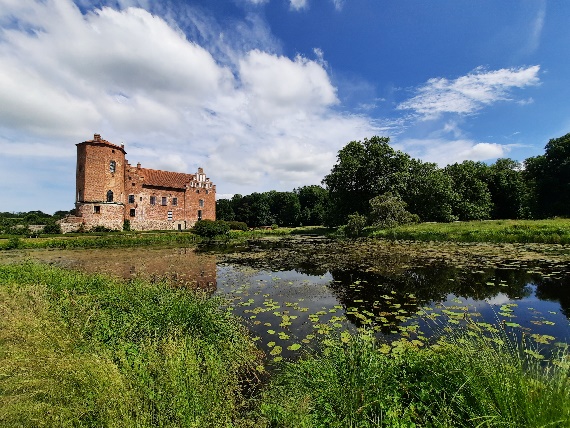 | 32 |
| Sweden | Vallkarra | High | 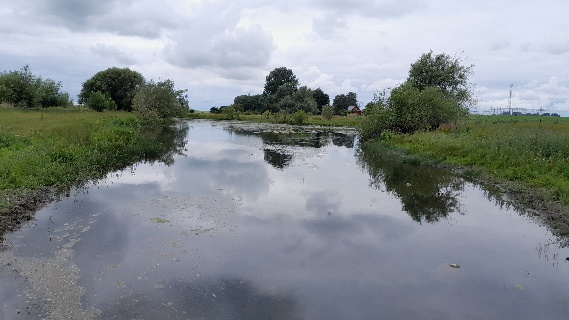 | 24 |

|  | **GPS coordinates** | |  |  |  |  |
| --- | --- | --- | --- | --- | --- | --- |
| **Locality** | **Lat.** | **Long.** | **Surface (m^2^)** | **Impervious surface (%)** | **Tree cover (%)** | **Genetic diversity** |
| Niepolomice | 50.10875 | 20.348707 | 7 710 | 0.00 | 0 | 0.164 |
| Zagorze | 50.083352 | 19.39736 | 5 959 | 0.37 | 15 | 0.166 |
| Torups | 55.564293 | 13.205614 | 9 639 | 1.35 | 15 | / |
| Se_S_R2 | 55.576 | 13.211 | / | / | / | 0.161 |
| Vallkarra | 55.738166 | 13.153274 | 4 402 | 0.19 | 5 | 0.187 |

**Table S2.** Spearman correlation analysis for each latitude separately between the five phenotypic traits: mass, developmental time (dev. time), head width, growth rate based on mass (GRM) and wing pad length.

| Central latitude | Mass | Dev time | Head width | GRM | Wing pad |
| --- | --- | --- | --- | --- | --- |
| Mass | 1 |  |  |  |  |
| Dev. time | 0.17 | 1 |  |  |  |
| Head width | **0.64***** | -0.01 | 1 |  |  |
| GRM | **0.27*** | **-0.86***** | 0.28 | 1 |  |
| Wing pad | **0.53***** | 0 | **0.42***** | **0.23*** | 1 |

| High latitude | Mass | Dev time | Head width | GRM | Wing pad |
| --- | --- | --- | --- | --- | --- |
| Mass | 1 |  |  |  |  |
| Dev. time | **0.39***** | 1 |  |  |  |
| Head width | **0.79***** | **0.39***** | 1 |  |  |
| GRM | -0.17 | **-0.95***** | **-0.22*** | 1 |  |
| Wing pad | **0.73***** | 0.16 | 0.79 | 0.01 | 1 |

**Fig. S1.** Weekly temperatures for each Polish (PL; central latitude) and Swedish (SW; high latitude) pond. A) temperature was measured with dataloggers placed at a depth of ca. 40 cm during the year 2022 – 2023 and B) temperature was estimated using Flake (Flake 2009) over the period 1998 – 2009. In A) for Zagorze pond we placed two dataloggers at different locations in the pond: logger 1 in a partly shaded area and logger 2 in a sun-exposed area.

**
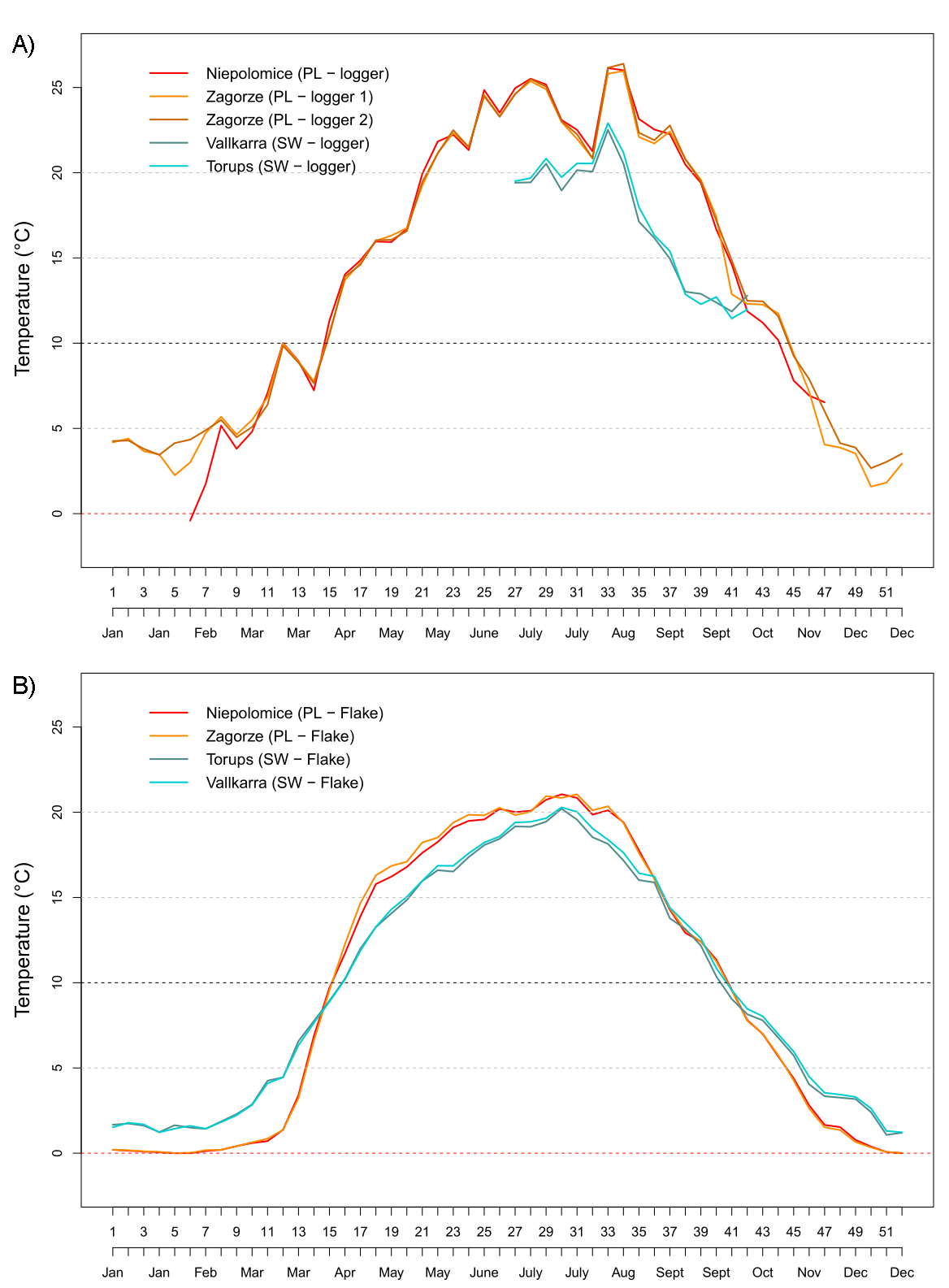
**

**Fig. S2.** Principal component analysis (PCA) plots with the phenotypic data showing differences in the four phenotypic variables for the two central- (Zagorze and Niepolomice) and high- (Vallkarra and Torups) latitude ponds.

**
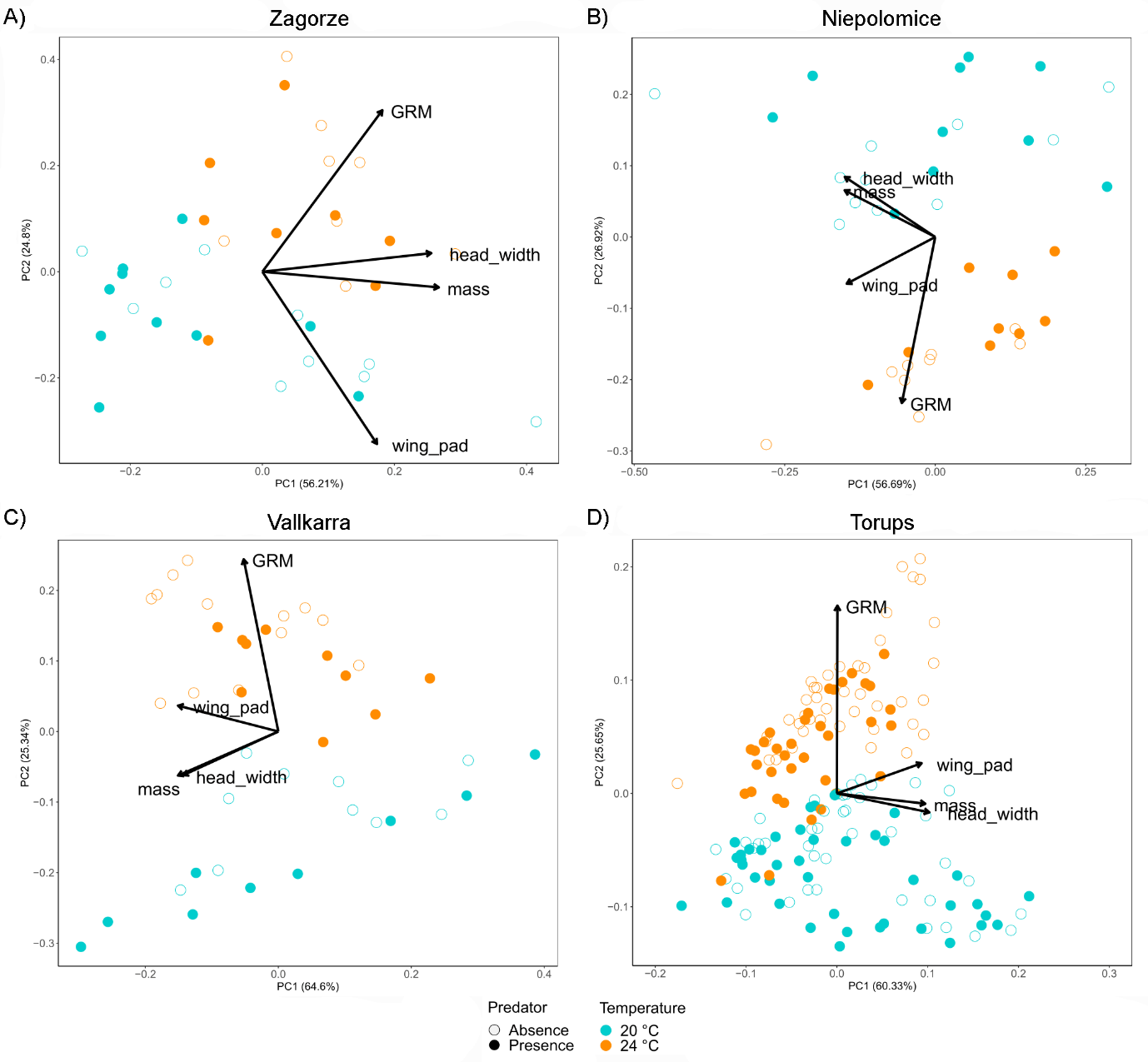
**

**Fig. S3.** Principal component analysis (PCA) plots with the transcriptomic data for the two central- (Zagorze and Niepolomice) and high- (Vallkarra and Torups) latitude ponds.

**
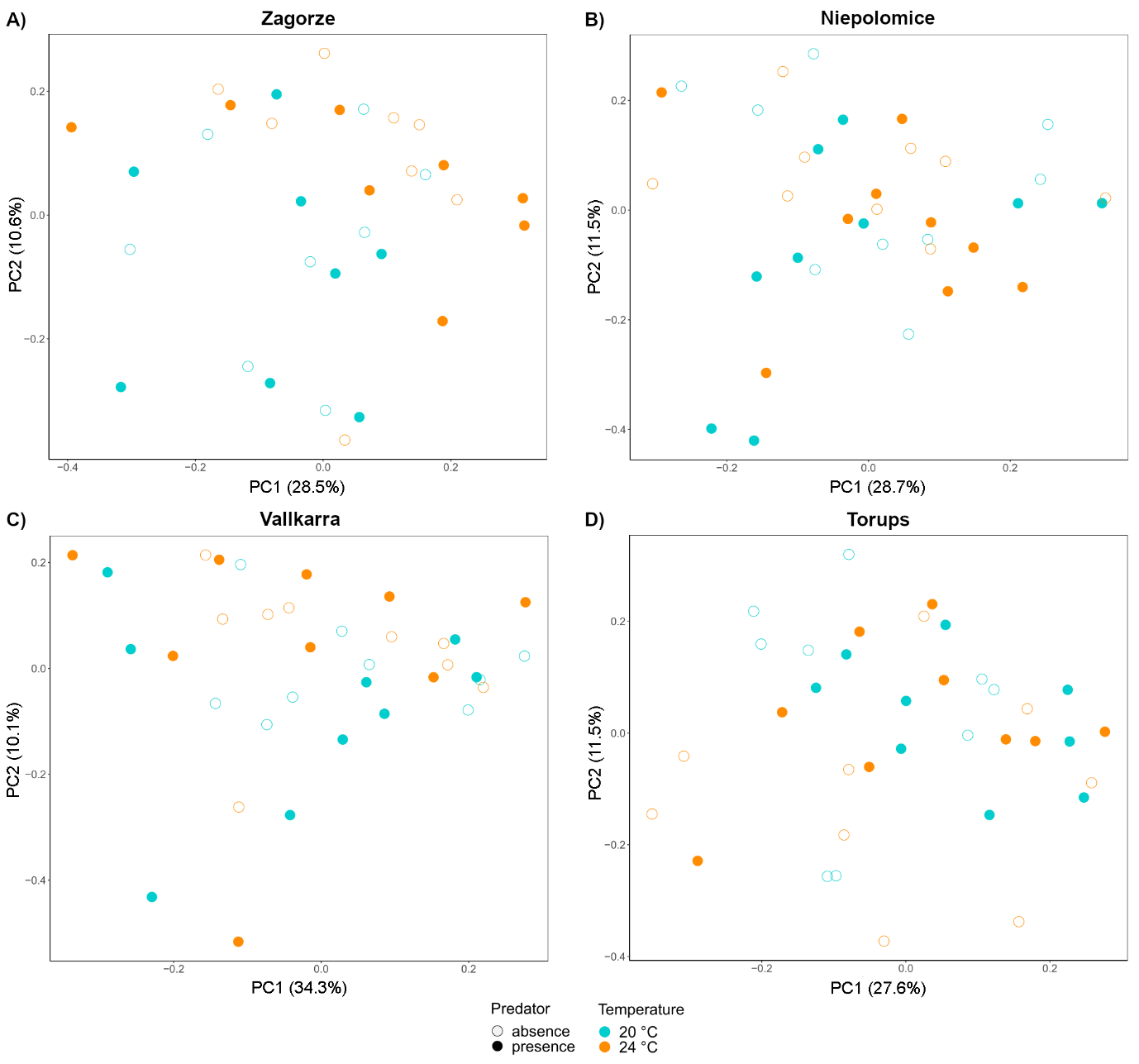
**

**Fig. S4**. Transcript per million (TPM) values for the gene *SALIVARY GLUE PROTEIN SGS-3-LIKE* (LOC124163034) showing higher expression at 20 °C in each pond.


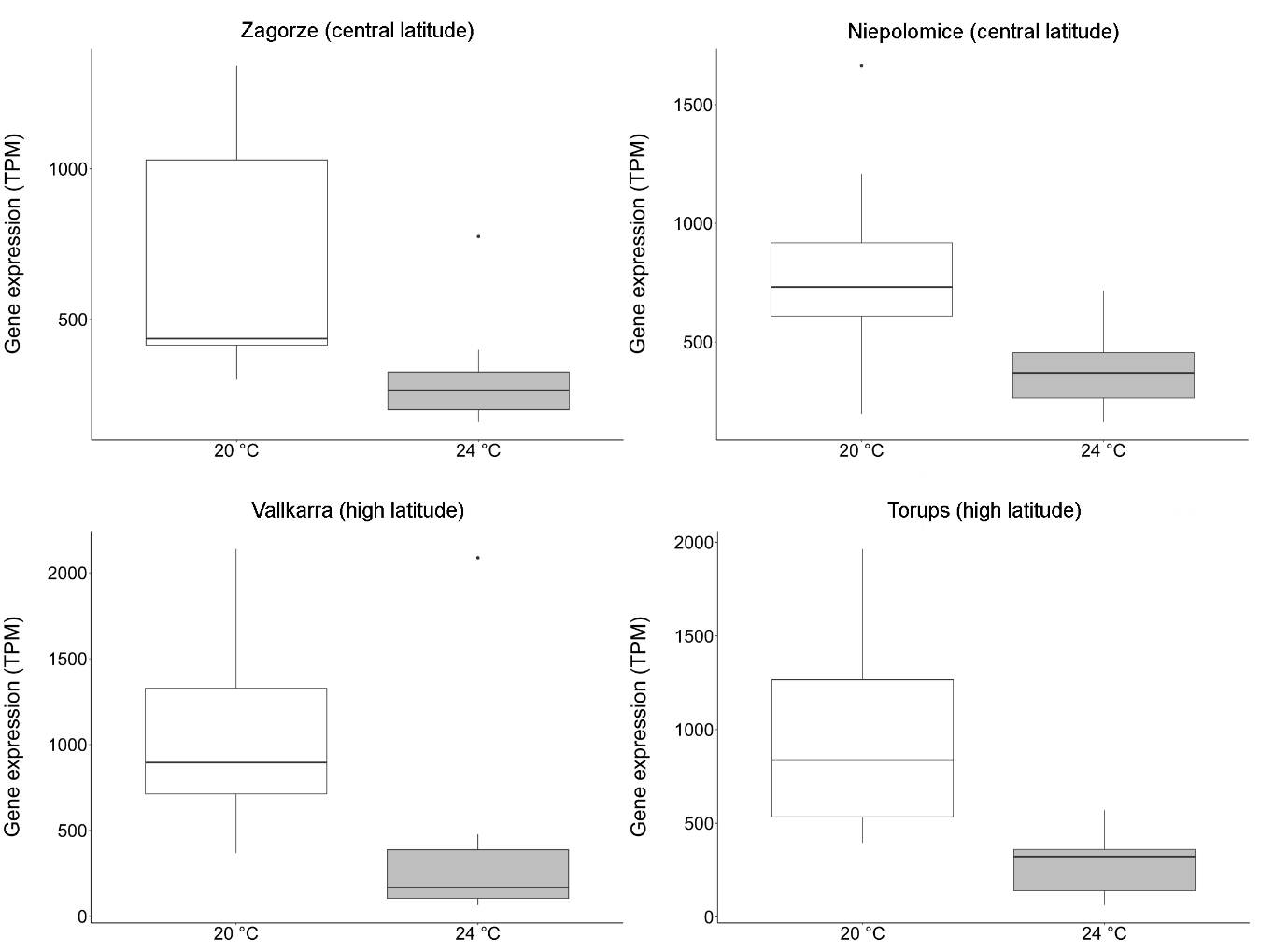


**Fig. S5**. Transcript per million (TPM) values for the gene *AGRIN* (LOC124156371) showing higher expression in the absence of a predator cue in two ponds.


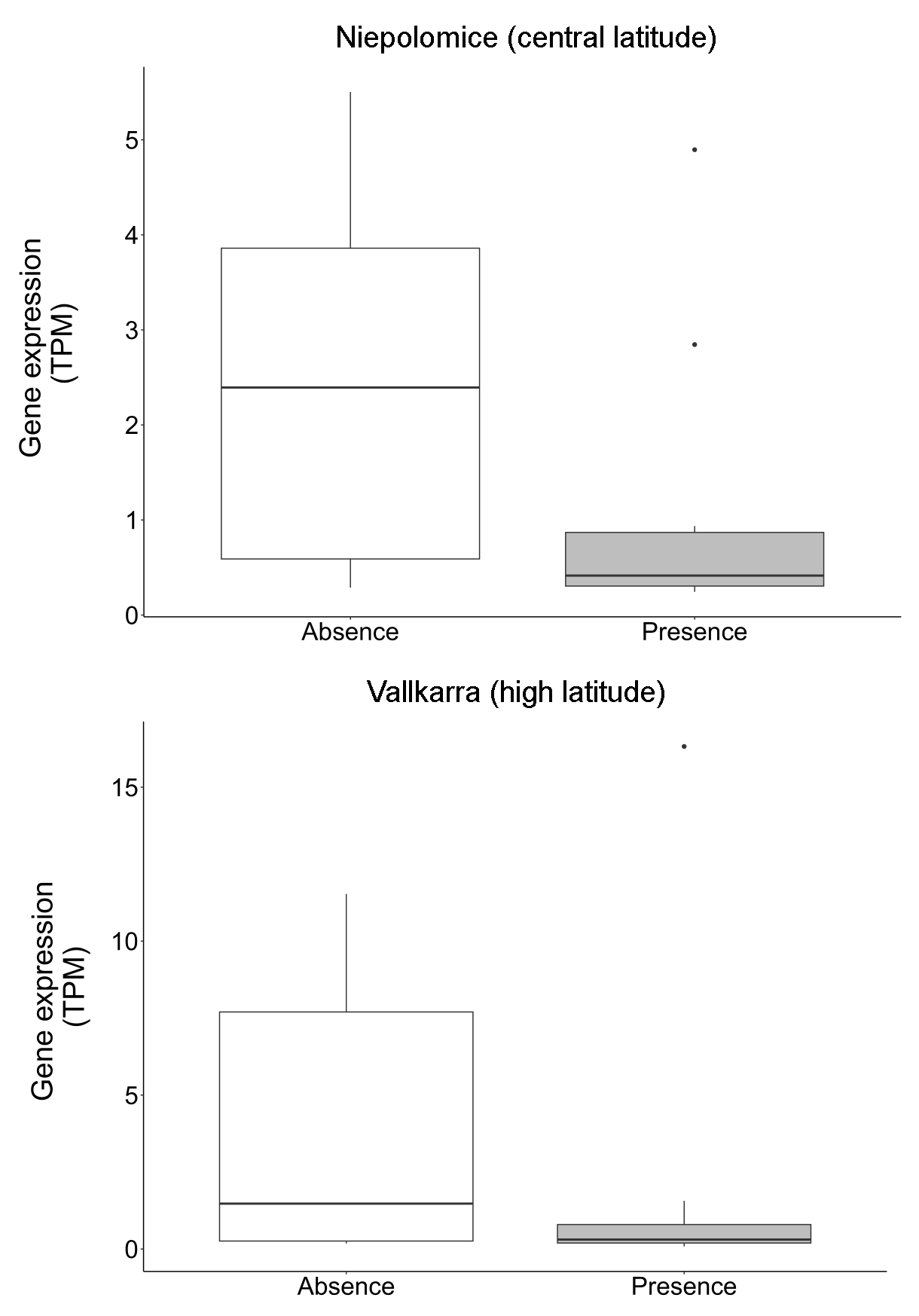


**Fig. S6**. Transcript per million (TPM) values for the gene *A-KINASE ANCHOR PROTEIN 14-LIKE* (LOC124169622) showing significant temperature × predator interaction in three ponds.


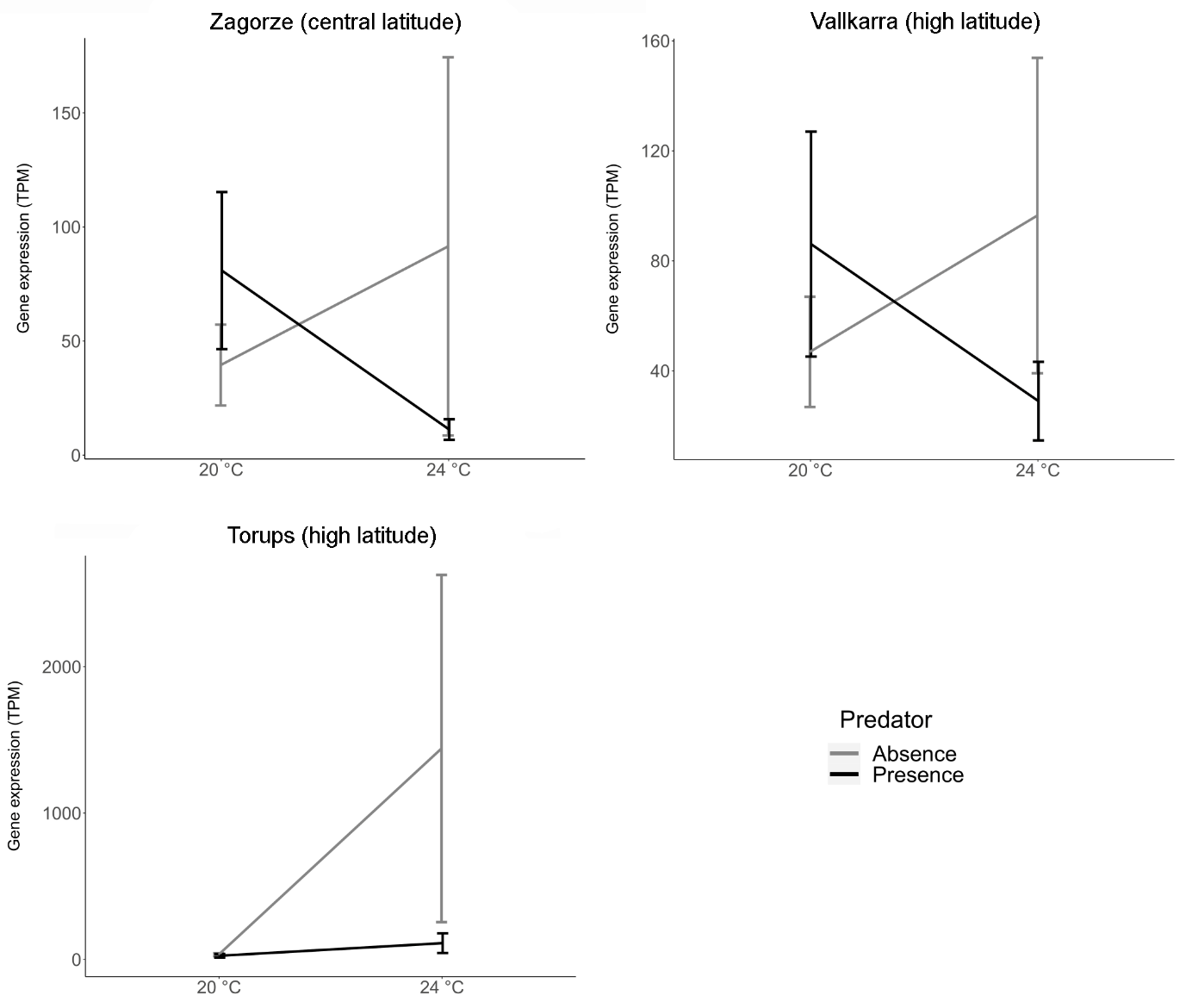


**Fig. S7**. Number of gene ontology (GO) terms that overlap across ponds in response to A) temperature, B) predator cue and C) temperature × predator cue (T × P). On the x-axis, green dots indicate the ponds being compared and values on the right side indicate the total number of GO terms affected by each treatment in each pond. Significant overlap across ponds is indicated by *** p < 0.001, ** p < 0.01; detailed results of the tests are presented in Table S8.


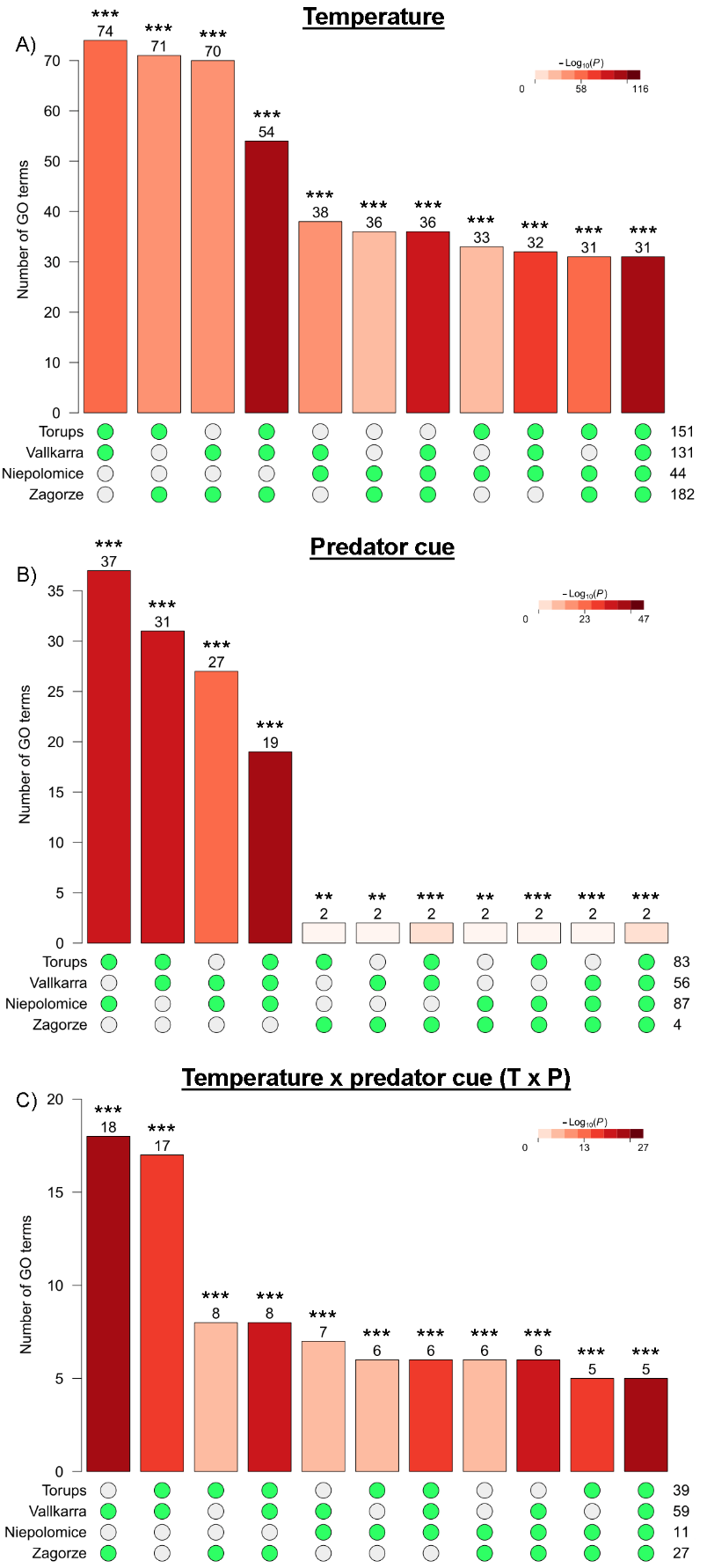

Supplement: Supplementary file 1 — File S1. Summary of the different steps of the growth chamber experiment. Figure S1. Weekly temperatures in each pond. Figure S2. Principal component analysis with the phenotypic data. Figure S3. Principal component analysis with the transcriptomic data. Figure S4. Expression values for the gene SALIVARY GLUE PROTEIN SGS‐3‐LIKE. Figure S5. Expression values for the gene AGRIN. Figure S6. Expression values for the gene A‐KINASE ANCHOR PROTEIN 14‐LIKE. Figure S7. Overlap in gene ontology terms. Table S1. Information about sampling sites. Table S2. Spearman correlation analysis. [file EVA-17-e70002-s005.docx]
